# Polycaprolactone-Based Mimetic Antimicrobial Peptide Copolymers Vesicles as an Effective Drug-Carrier for Cancer Therapy

**DOI:** 10.3390/polym11111783

**Published:** 2019-10-31

**Authors:** Yusheng Qian, Xinyu Zhou, Jing He, Chuncai Zhou

**Affiliations:** 1 School of Materials Science and Engineering, Tongji University, 4800 Caoan Road, Shanghai 201804, China; 1651601@tongji.edu.cn (Y.Q.); 1631451@tongji.edu.cn (X.Z.); 2 Department of Anatomy and Neurobiology, Tongji University School of Medicine, Shanghai 201804, China

**Keywords:** antibacterial peptide, amphiphilic copolymer, self-assembly, vesicle, drug delivery

## Abstract

A novel series of amphiphilic mimicking antimicrobial peptide copolymers PCL_16_-*b*-K_n_ can assemble in water to form uniform vesicles. Transmission electron microscopy was used to observe the vesicular structure of the nanoparticles, and dynamic light scattering revealed their uniform size and narrow dispersion. Critical vesiculation concentrations were also tested, revealing that these vesicles can exist at low concentrations. Furthermore, in vitro and intracellular drug release of doxorubicin(DOX)-vesicles were conducted. These vesicles could encapsulate DOX and achieve efficient intracellular drug release. Overall, these copolymer vesicles exhibit potential application value as multifunctional drug-carrier systems with antibacterial capability in cancer therapy.

## 1. Introduction

Cancer is one of the most important and deadly diseases worldwide [1,2]. Reports from the World Health Organization show that almost 1 in 6 deaths worldwide in 2018 were due to cancer. Chemotherapy is a common approach for cancer treatment; it uses drugs to kill cancer cells [1,2,3]. However, various limitations affect the efficiency of chemotherapy. For example, most chemotherapeutics possess poor aqueous solubility, harm normal cell toxicity due to the lack of selectivity, and cause multidrug resistance [4,5]. To handle these shortcomings, researchers have proposed nano-particles as novel drug carriers to help patients cope with cancer [6,7,8]. Nanoparticles, like vesicles [9,10,11], nanotubes [6,7,8], micelles [12,13,14], nanofibers [15,16,17], and nanosheet [18,19] are typically commonly formed by amphiphilic copolymers via self-assembly. Nanoparticles can encapsulate drugs via physical entrapment or solubilization to achieve various advantages, including controlled drug release, decreased cytotoxicity, increased aqueous solubility, and targeted action site [4,20,21,22,23]. Thus, nanoparticles have opened the door for developing effective drug delivery schemes and have shown substantial potential for applications in life-threatening disease treatments, such as cancer therapy.

Chemotherapy usually causes a decline in immunity, causing cancer patients to suffer from bacterial infection or threatening their lives. Therefore, avoiding bacterial infection is essential during cancer therapy. As a kind of novel antibacterial agent, antimicrobial peptides (AMPs) have gained increasing attention over the past few decades. AMPs exhibit excellent antibacterial capability and a unique antibacterial mechanism [24,25]. AMPs achieve their bactericidal effect by physical disruption of bacterial membranes; however, inducing bacterial drug resistance presents difficulty [26]. Research illustrates that such a unique antibacterial mechanism is relevant to specific AMP structures, including cationic moieties such as lysine and hydrophobic moieties such as phenylalanine [27,28]. Lots of AMPs are amphiphilic peptides, namely, they are composed of hydrophobic and hydrophilic residues. AMP-nanoparticles can be prepared in an aqueous solution through self-assembly of these AMPs. AMP assemblies are ideal drug delivery systems exhibiting both drug release and antibacterial capabilities during cancer treatment. Our group has reported several amphiphilic block AMPs containing lysine and phenylalanine [29,30]. These AMPs exhibit high bactericidal efficiency and low cytotoxicity. Vesicles formed by these AMPs have good performance when carrying drugs [31].

Motivated by the excellent properties and promising applications of AMP nanoparticles, synthetic amphiphilic AMP–mimetic polymers are proposed [32,33,34]. These amphiphilic copolymers stimulate the structural features of AMPs, that is, cationic and hydrophobic moieties. Amphiphilic copolymers not only possess good properties, such as a unique bactericidal mechanism, the capability for self-assembly into nanoparticles that are similar to those of natural AMPs, but also show more convenient synthesis strategy than natural AMPs. In previous work, Zhou et al. have reported the minimal inhibition concentration (MIC) of amphiphilic copolymers was less than 32 μg/mL against both Gram-negative and Gram-positive bacteria [35]. The broad antibacterial capability has been proved, which will play a vital role in cancer therapy.

In our previous work, a series of amphiphilic mimicked AMP copolymers, PCL_16_-*b*-K_n_, which can inhibit both Gram-negative and Gram-positive bacteria. In the present work, we studied the self-assembling behavior of these block copolymers in aqueous solutions. The morphologies and sizes of nanoparticles were characterized by transmission electron microscopy (TEM) and dynamic light scattering (DLS), respectively. Critical vesiculation concentrations (CVCs) were also studied. Drug delivery potential was investigated by in vitro and intracellular doxorubicin (DOX)-release experiments. Based on the results, amphiphilic diblock copolymers PCL_16_-*b*-K_n_ could serve as efficient drug carriers for cancer treatment. Given the excellent antibacterial properties and low cytotoxicity of PCL_16_-*b*-K_n_ in previous works, such diblock copolymer nanoparticles can be ideal “armed” drug delivery systems for applications in nanomedical fields.

## 2. Experimental

### 2.1. Materials

The amphiphilic diblock copolymers PCL_16_-*b*-K_n_ were synthesized via facile coupling reaction in our previous works [36]. Tetrahydrofuran (THF), acetone, tris buffer (0.01 M, pH 7.4) were obtained from Tansoole Co. Ltd. (Zhuhai, China). Pyrene and doxorubicin hydrochloride (DOX·HCl) was received from Adamas Reagent Co. Ltd. (Shanghai, China). Dulbecco’s modified Eagle’s medium (DMEM) was supplied from Hyclone and fetal bovine serum (FBS) was bought from Gibco (Shanghai, China). Human HCC cell (SMMC-7721) and normal liver cell (HL-7702) were supplied by the Shanghai Institute of Biochemistry and Cell Biology (Shanghai, China).

### 2.2. Preparation of PCL_16_-b-K_n_ Copolymer Vesicles

5.000 mg PCL_16_-*b*-K_n_ diblock copolymers were dissolved in 2.0 mL THF/H_2_O (1:1, *v*:*v*). Additionally, 4.0 mL deionized water was added dropwise to the solution during 10 min with continuous stirring. The solution was stirred for 12 h. After that, the solution was dialyzed in a dialysis tube (3500 MWCO) against deionized water for 24 h (renewing the deionized water every 2 h) to achieve diblock copolymer vesicles.

### 2.3. Critical Vesiculation Concentration (CVC) Test

Critical vesiculation concentration (CVC) is defined as the lowest concentration that copolymers can self-assemble into nanoparticles in solution. 25 mL pyrene/acetone solution which has a concentration of 0.12 mg/mL was prepared to detect self-assembly. Adding 10 μL of pyrene/acetone solution to centrifuge tubes made acetone evaporate. Different concentrations (1000, 500, 250, 125, 64, 32, 16, 8, 4, 2, and 1 μg mL^−1^) of PCL_16_-*b*-K_n_ solutions were configured via serial 2-fold dilution and was transfered to glass bottles, while remaining contantly whisked for 12 h. The final values were tested and caculated in the same way as in the reported literature [3].

### 2.4. In Vitro DOXencapulation and Release

The anticancer drug encapulation experiment was carried out based on the literature protocol [37] and modified as follows: DOX·HCl (3.7 mg) and PCL_16_-*b*-K_20_ copolymers (12.7 mg) was dissolved in THF (2.0 mL) and then deionized water (10.0 mL) was dropped into the copolymer/DOX mixture solution within 20 min to form DOX-loaded PCL_16_-*b*-K_20_ vesicles. The vesicles’ solution was stirred overnight to reach equilibration. After that, the DOX-loaded PCL_16_-*b*-K_20_ vesicle solution was purified by using a dialysis tube (14000 MWCO, Union Carbide Corporation, Danbury, CT, USA) against 1000 mL deionized water at 25 °C for 3 h to remove THF and unloaded free drug (renew water every 30 min). Prepared DOX-loaded PCL_16_-*b*-K_20_ vesicle solutions were divided into three new dialysis tubes (each tube with 3.0 mL of solutions) and each tube was dialyzed in a breaker against in 80 mL of tris buffer (0.01M; pH 7.4) at 37 °C at 190 revolutions per minute. The DOX concentration in the breaker was detected by fluorescence spectroscopy (excitation at 461 nm and emission at 591 nm, Thermo Scientific, Vancouver, BC, Canada) every time unite to draw the cumulative release curve. The drug loading efficiency (DLE) and drug loading content (DLC) were evaluated by fluorescence spectroscopy (excitation at 461 nm and emission at 591 nm) according to a calibration curve of aqueous DOX solution with known concentrations.

The formulas to calculate the drug loading efficiency (DLE) and drug loading content (DLC) were presented as follows:

(1) $$\mathrm{DLC}\%=\frac{\mathrm{weight}\;\mathrm{of}\;\mathrm{drug}\;\mathrm{encapsulated}\;\mathrm{in}\;\mathrm{copolymer}}{\mathrm{weight}\;\mathrm{of}\;\mathrm{copolymer}}\times 100\%$$

(2) $$\mathrm{DLE}\%=\frac{\mathrm{weight}\;\mathrm{of}\;\mathrm{drug}\;\mathrm{encapsulated}\;\mathrm{in}\;\mathrm{copolymer}}{\mathrm{weight}\;\mathrm{of}\;\mathrm{drug}\;\mathrm{in}\;\mathrm{feed}}\times 100\%$$

A control release curve of pure DOX·HCl was also evaluated. The solution of DOX·HCl in 10.0 mL of deionized water (0.37 mg/mL) was equally divided into three dialysis tubes. Each tube was dialyzed against 80.0 mL of tris buffer (0.01M; pH 7.4) in a breaker at 37 °C, stirring at revolutions per minute. DOX concentration in the breaker was determined using the similar method stated above. The expriments stated above were all repeated three times.

### 2.5. Characterization

*Dynamic Light Scattering.* The DLS of PCL_16_-*b*-K_n_ was tested by Nano-ZS 90 Nanosizer (Malvern Instruments, Shanghai, China). Each example was tested with three replication. The computed diffusion coefficients measured by DLS and combined with the Stokes-Einstein equation were used to calculate the particle diameter and the size distribution.

*Transmission Electron Microscopy (TEM).* TEM images of copolymer PCL_16_-*b*-K_n_ vesicle was used for the reported protocol using a JEOL JEM 2100F instrument (JEOL instrument, Tokyo, Japan) [35].

Fluorescence Spectroscopy. The measurement of Vesicles‘ fluorescence intensities was carried out with fluorescence spectrometer via a Lumina Fluorescence Spectrometer (Thermo Scientific, Vancouver, Canada).

### 2.6. Intracellular Release of DOX-Loaded PCL_16_-b-K_20_ Nanoparticles

The intracellular drug release experiment was developed based on the literature [38,39]. Cancer cells (SMMC-7721 cells) and normal human liver-derived cells (MIHA cells) were chosen to detect the intracellular release of drug-loaded PCL_16_-*b*-K_20_ vesicles using confocal laser scanning microscopy (CLSM). First, SMMC-7721 cells and MIHA cells were separately cultured in 96-well plates with Dulbecco’s modified Eagle’s medium (DMEM) and 10% fetal bovine serum (FBS) for 24 h at 37 °C. Meanwhile, DOX-loaded PCL_16_-*b*-K_20_ vesicles at a concentration of 5000 μg mL^−1^ were prepared through similar steps to those mentioned above. Afterwards, 480 μL of fresh DMEM with 10% FBS replaced the culture media after finishing the cell culture. Then, 20 μL of DOX-loaded vesicles were added and the mixtures were incubated at 37 °C in a humidified 5% CO_2_-containing atmosphere for 4 h. After eliminating the culture media, both cells were washed three times with phosphate buffered saline (PBS). Finally, both cells were fixed with 4% paraformaldehyde and were pictured by confocal laser scanning microscopy (CLSM) to observe intracellular DOX release.

In addition, cell viability of cancer cells and normal cells with DOX-loaded vesicles were measured by CCK-8. Cancer cells (SMMC-7721 cells) and normal human liver-derived cells (MIHA cells) were cultured by similar methods to those mentioned above. After culturing for 24 h, the cells were separately mixed with DOX-loaded vesicles at different concentrations (62, 125, 250, 500, or 1000 μg mL^−1^). The mixtures were incubated for 1d, 2d, and 3d. Then, the cell viability of all samples was detected via CCK-8. The dye was mixed with cells and incubated for another 1 h. The absorbance was measured at 450 nm and 630 nm by multimode plate reader. The calculation of relative cell viability (% control) was the comparison of the absorbance at 450 nm with controls containing only cell culture medium. Six groups of replicates were carried out to characterize the cytotoxicity of the vesicles.

## 3. Results and Discussion

### 3.1. Synthesis of PCL_16_-b-K_n_ Copolymers

**Scheme 1:**
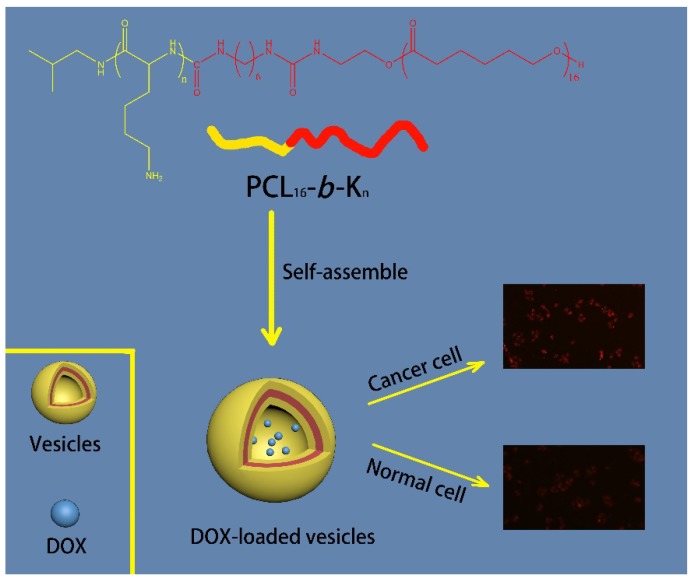
PCL_16_-*b*-K_n_ vesicle drug delivery system for cancer therapy.

The synthesis of the PCL_16_-*b*-K_n_ diblock copolymers has been introduced in our previous published work [35]. In brief, polylysine blocks were prepared by NCA ring-opening polymerization. PCL-NH_2_ was also prepared by ring-opening polymerization of caprolactone. A coupling reaction using hexamethylene diisocyanate (HDI) connected polylysine, PCL, and block copolymers was obtained. The structure of the PCL_16_-*b*-K_n_ diblock copolymers and the graphic abstract of this work were shown in the Scheme 1.

### 3.2. Self-Assembly of PCL_16_-b-K_n_ Copolymers

**Figure 1:**
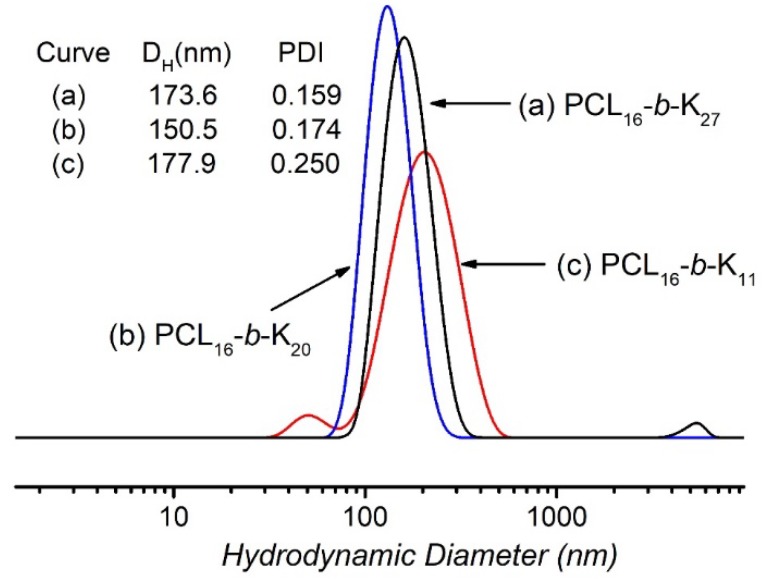
DLS study of (**a**) PCL_16_-*b*-K_27_, (**b**) PCL_16_-*b*-K_20_, (**c**) PCL_16_-*b*-K_11_diblock copolymer vesicles.

Amphiphilic PCL_16_-*b*-K_n_ diblock copolymers form nanoparticles in Tetrahydrofuran (THF)/H_2_O (1:4, *v*:*v*) via self-assembly. PCL_16_, forms the vesicle membrane as a hydrophobic chain, whereas polylysine hydrophilic moieties form the corona. The sizes and morphologies of vesicles were tested and confirmed by DLS and TEM, respectively. Figure 1 shows the DLS results. The average hydrodynamic diameters (Dh) and polydispersity index (PDI) of three copolymer vesicles, namely, PCL_16_-*b*-K_11_, PCL_16_-*b*-K_20_, and PCL_16_-*b*-K_27_, reached 173.6, 150.5, and 177.9 nm and 0.159, 0.174 and 0.250, respectively. These results confirmed the narrow PDI and similar sizes of these vesicles, indicating their good dispersion in aqueous solutions.

**Figure 2:**
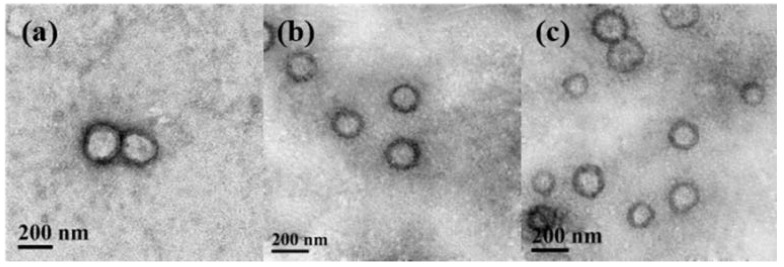
Transmission Electron Microscopy (TEM) images of (**a**) PCL_16_-*b*-K_11_, (**b**) PCL_16_-*b*-K_20_, (**c**) PCL_16_-*b*-K_27_ diblock copolymer vesicles.

TEM was used to observe the exact structures of three block copolymers (PCL_16_-*b*-K_11_, PCL_16-_*b*-K_20_, and PCL_16_-*b*-K_27_). Figure 2 shows the TEM images. All TEM images demonstrated the hollow structure of nanoparticles, corresponding to the morphologies of vesicles. According to the TEM images, the sizes of these vesicles approximated 180 nm, which is reasonably consistent with DLS results. Considering both DLS and TEM results, PCL_16_-*b*-K_n_ copolymers can self-assemble into uniform-size vesicles. The vesicular structure could confer these copolymers with potential application as drug carriers.

**Figure 3:**
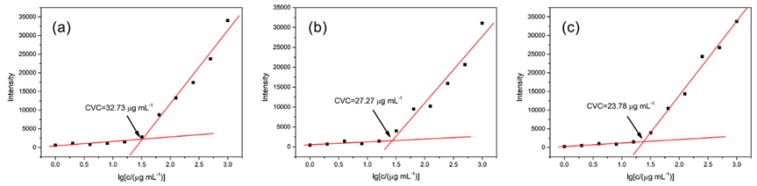
Determination of the Critical Vesiculation Concentration (CVC) of (**a**) PCL_16_-*b*-K_11_, (**b**) PCL_16_-*b*-K_20_, (**c**) PCL_16_-*b*-K_27._

The definition of CVC is the lowest concentration of solutions needed to form stable vesicles. Pyrene served as a probe to measure the CVCs of PCL_16_-*b*-K_n_ copolymers. Figure 3 revealed that the CVCs of PCL_16_-*b*-K_11_, PCL_16-_*b*-K_20_, and PCL_16_-*b*-K_27_ reached 32.73, 27.27, and 23.76 μg mL^−1^, respectively. As the length of polylysine blocks increased, the CVCs exhibited a decreasing trend. PCL_16_-*b*-K_n_ copolymers can self-assemble into vesicles in an aqueous solution at low concentrations.

Compared with the lowest bactericidal concentration of three diblock copolymers, almost all PCL_16_-*b*-K_n_ diblock copolymers possessed higher CVC values than the lowest bactericidal concentrations reported in our previous work [35]. Such a result indicated that copolymer vesicles tended to disassemble into individual chains when acting on the bacterial membrane. This may be due to the unique pore-forming antibacterial mechanism, and individual chains were more favorable than vesicular structures to enable hydrophobic segments to be bent and twisted into bacterial membranes. While PCL_16_-*b*-K_n_ diblock copolymers work as a drug dilivery system, they can also achieve the purpose of sterilization to improve the immunity of cancer patients. Therefore higher CVC values can increase the effectiveness of cancer treatments.

### 3.3. In Vitro DOX Encapulation and Release

**Figure 4:**
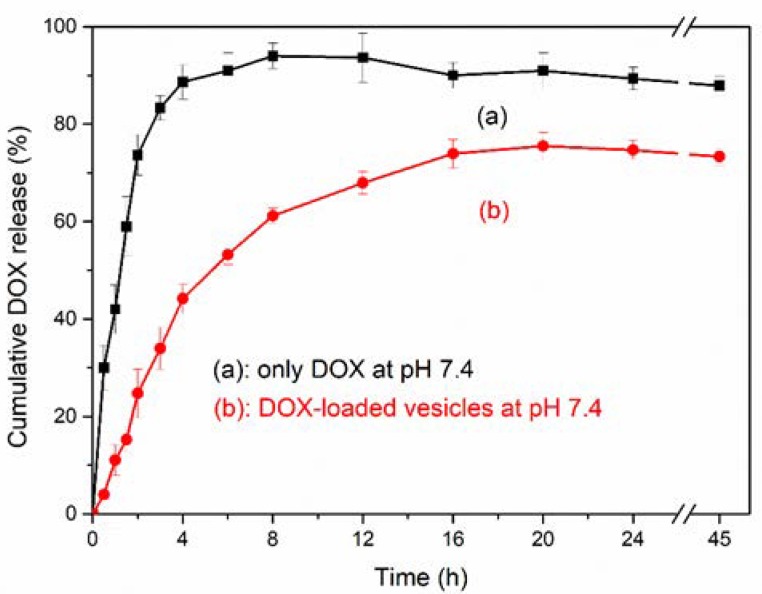
DOX release profiles of (**a**) free DOX and (**b**) DOX-loaded vesicles in 0.01 M tris buffer at pH 7.4.

PCL_16-_*b*-K_20_ were used to undergo in vitro and intracellular drug release experiment according to our previous work because PCL_16_-*b*-K_20_ have better properties (e.g., antibacterial ability) [36]. The drug loading efficiency (DLE) and drug loading content (DLC) of copolymer vesicles were 9.69% and 33.27%, respectively. Figure 4b displayed the in vitro drug release curve. The control experiment with free DOX alone (Figure 4a) indicated rapid drug elution. 60% of DOX was released in the first 2 h, and the cumulative release rate reached 90% after 4 h. Unlike the free DOX, the DOX-loaded copolymer vesicles showed a lower initial release rate. Only about 25% of DOX was released within 2 h, and DOX was continuously and slowly released within 20 h. These results proved that the copolymer vesicles can realize remarkable drug sustained-release with less waste of the drug. Thus, they can be used as antibacterial drug carriers, and exert multiple effects such as antibacterial and drug sustained-release in the context of anticancer treatment.

Five kinetic models including Zero-order, First-order, Higuchi, Hixson-Crowell, and Korsmeyer-Peppas were used to fit the drug release curve [40]. The formulas and the determination coefficients (r^2^) of different models were shown in Table 1. By comparing the determination coefficients, it can be revealed that the drug release process of vesicles formed by PCL_16_-*b*-K_20_ diblock copolymers obeyed the first-order models because its determination coefficient was greater than 0.994. Such a result suggested that the decisive factor of controlled release in PCL_16_-*b*-K_20_ vesicles is the internal and external concentration difference. The release speed reduced gradually, so the burst release phenomenon could not be detected [41]. Additionally, the first-order kinetic models usually fitted the drug release process of porous materials [42], so the coronas of the nanoparticles formed by hydrophilic poly-lysine may present good penetrating ability, especially for DOXs.

### 3.4. Intracellular Drug Release of DOX-loaded PCL_16_-b-K_20_ Vesicles

**Figure 5:**
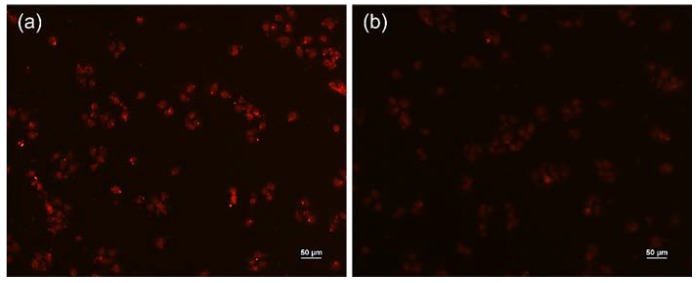
(**a**) The CLSM image of intracellular drug release in cancer cells; (**b**) The CLSM image of intracellular drug release in normal cells.

To explore the drug release efficiency against cancer cells, we selected cancer (SMMC-7721 cells) and normal human liver-derived cells (minor histocompatibility antigen cells) as experimental cells, and confocal laser scanning microscopy (CLSM) was used to present the intracellular drug release behavior of DOX-loaded PCL_16_-*b*-K_20_ vesicles. Cultured cancer cells were incubated with DOX-loaded vesicles, and CLSM image was obtained after washing and fixing the cells. Figure 5 presents the CLSM images of intracellular drug release of DOX-loaded vesicles. Figure 5a,b show the CLSM images of cancer and normal cells, respectively. DOX fluorescence was evidently observed in cancer cells (red signal), whereas weakened fluorescence was noted in normal cells. The results indicate that DOX-loaded PCL_16_-*b*-K_20_ vesicles could target and act on cancer cells. The findings further confirmed that such vesicles could release DOX in cancer cells and potentially serve as effective drug carriers for cancer cells.

### 3.5. CCK-8 Test of DOX-Loaded PCL_16_-b-K_20_ Vesicles

**Figure 6:**
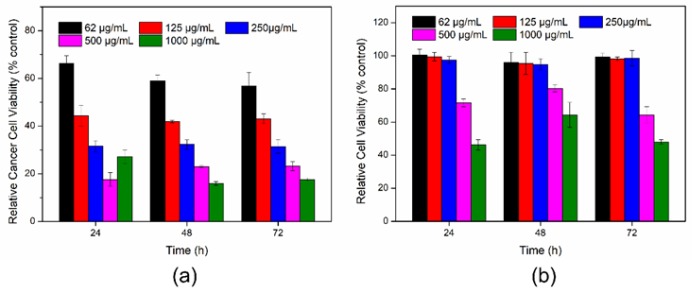
(**a**) Relative cancer cell viability with DOX-loaded PCL_16_-*b*-K_20_ vesicles; (**b**) relative normal cell viability with DOX-loaded PCL_16_-*b*-K_20_ vesicle (*n* = 6).

Cell viability with DOX-loaded vesicles was measured via CCK-8 to evaluate their killing capability against cancer and normal cells. Cultured cancer and normal cells were incubated with certain DOX-loaded vesicles. Cell viability was then detected through a CCK-8 method for 24, 48, and 72 h. Figure 6a,b showed the results regarding cancer and normal cells, respectively. Cancer cell viability exhibited an evident reduction in the presence of DOX-loaded polymer vesicles. Even at low concentrations (62 μg mL^−1^) of DOX-loaded vesicles, cancer cell viability was lower than 60%. And as the concentration of DOX-loaded vesicles increased, the cell viability of cancer cells continued to decrease significantly. By contrast, normal cell viability was maintained by more than 95% with DOX-loaded vesicles at 250 μg mL^−1^ concentration, revealing that DOX-loaded vesicles exhibited low cytotoxicity against normal cells. The results confirmed that DOX-loaded vesicles possessed high selectivity to cancer cells and could achieve an effective killing capability against cancer cells with a negligible effect towards normal cells.

Overall, both LSCM and CCK-8 results verified that DOX-loaded copolymer vesicles can effectively target cancer cells, and also improve the efficiency of anticancer drugs and reduce their toxicity to normal cells. This may be because the surface of the cancer cell membrane possesses more negative charges than normal cells [43]. Kim et al. reported nanoparticles processing different cytotoxicity to cancer cells and normal cells [44]. They also reported that the interactions between the membrane and the vesicles were mainly affected by electrostatic and hydrophobic properties. Thus, the selectivity mechanism of PCL_16_-*b*-K_20_ vesicles was similar to the process they mentioned. Compared with normal cells, PCL_16_-*b*-K_20_ vesicles can be absorbed onto the surface of cancer cells more easily via electrostatic interaction. Afterwards, the drug carriers are swallowed by cancer cells.

The immune ability of patients after chemotherapy is very low and antibiotics are usually required. However, traditional antibiotics may lead to serious side-effects for patients who have undergone chemotherapy. In addition, the abuse of antibiotics has led to the emergence of more drug-resistant pathogens. Compared with traditional antibiotics, AMPs can kill bacteria effectively and will not induce resistance due to its special antibacterial mechanism. Compared with reported drug deliver nanoparticles, PCL_16_-*b*-K_n_ copolymers vesicles can not only be used as a drug carrier, but also can be used as a novel antibiotic to inhibt bacterial infection.

## 4. Conclusions

In summary, PCL_16_-*b*-K_n_ copolymers mimicking AMPs not only exhibited broad antibacterial capability but could also self-assemble into vesicles, with hydrophilic polylysine and hydrophobic PCL forming the corona and membrane, respectively. The sizes and morphologies of vesicles were detected by DLS and TEM, respectively, confirming the formation of uniform vesicles. This kind of vesicles could encapsulate and release drugs. Drug-loaded vesicles could also achieve controlled intracellular drug release and effective killing capability against cancer cells with low cytotoxicity to normal cells. Therefore, the amphiphilic block copolymer vesicles could function as multifunctional drug carriers in nanomedicine for cancer therapy.

## Figures and Tables

**Table 1:** Mathematical models used for fitting the experimental data of vesicles formed by PCL_16_-b-K_20_ and different determination coefficients (r2).

Models	Formula	r2
Zero-Order	$Q_{t}=Q_{0}+K_{0}t$	0.6504
First-Order	$\log Q_{t}=logQ_{0}-\frac{K_{1}t}{2.303}$	0.9942
Hixson-Crowell	$\sqrt[{3}]{Q_{0}}-\sqrt[{3}]{Q_{t}}=K_{HC}t$	0.9925
Higuchi	$Q_{t}=K_{H}\sqrt[{2}]{t}$	0.9461
Korsmeyer-Peppas	$\frac{Q_{t}}{Q_{\infty }}=K_{KP}t^{n}$	0.9408

## References

[1] Bray F., Ferlay J., Soerjomataram I., Siegel R.L., Torre T.A., Jemal A. (2018). Global cancer statistics 2018: Globocan estimates of incidence and mortality worldwide for 36 cancers in 185 countries. CA Cancer J. Clin..

[2] Perez-Herrero E., Fernandez-Medarde A. (2015). Advanced targeted therapies in cancer: Drug nanocarriers, the future of chemotherapy. Eur. J. Pharm. Biopharm..

[3] Chabner B.A., Roberts T.G. (2005). Timeline—Chemotherapy and the war on cancer. Nat. Rev. Cancer..

[4] Estanqueiro M., Amaral M.H., Conceição J., Sousa Lobo J.M. (2015). Nanotechnological carriers for cancer chemotherapy: The state of the Art. Colloids Surf. B..

[5] Liu H.G., Lv L., Yang K. (2015). Chemotherapy targeting cancer stem cells. Am. J. Cancer Res..

[6] Kim C.J., Kurauchi S., Uebayashi T., Fujisaki A., Kimura S. (2017). Morphology change from nanotube to vesicle and monolayer/bilayer alteration by amphiphilic block polypeptides having aromatic groups at C terminal. Bull. Chem. Soc. Jpn..

[7] Sun J., Jiang X., Lund R., Downing K.H., Balsara N.P., Zuckermann R.N. (2016). Self-Assembly of crystalline nanotubes from monodisperse amphiphilic diblockcopolypeptoid tiles. Proc. Natl. Acad. Sci. USA.

[8] Burgess N.C., Sharp T.H., Thomas F., Wood C.W., Thomson A.R., Zaccai N.R., Brady R.L., Serpell L.C., Woolfson D.N. (2015). Modular design of self-assembling peptide-based nanotubes. J. Am. Chem. Soc..

[9] Song Z.Y., Kim H.J., Ba X.C., Baumgartner R., Lee J.S., Tang H.Y., Leal C., Cheng J.J. (2015). Polypeptide vesicles with densely packed multilayer membranes. Soft Matter..

[10] Song J.B., Yang X.Y., Jacobson O., Lin L.S., Huang P., Niu P., Ma Q.J., Chen X.Y. (2015). Sequential drug release and enhanced photothermal and photoacoustic effect of hybrid reduced graphene oxide-loaded ultrasmall gold nanorod vesicles for cancer therapy. ACS Nano.

[11] Ghanbarzadeh S., Khorrami A., Arami S. (2015). Nonionic surfactant-based vesicular system for transdermal drug delivery. Drug Deliv..

[12] Toughraï S., Malinova V., Masciadri R., Menon S., Tanner P., Palivan C., Bruns N., Meier W. (2015). Reduction-sensitive amphiphilic triblock copolymers self-assemble into stimuli-responsive micelles for drug delivery(a). Macromol. Biosci..

[13] Qiu H.B., Hudson Z.M., Winnik M.A., Manners I. (2015). Multidimensional hierarchical self-assembly of amphiphilic cylindrical block comicelles. Science.

[14] Vieira V.M.P., Liljeström V., Posocco P., Laurini E., Pricl S., Kostiainen M.A., Smith D.K. (2017). Emergence of highly-ordered hierarchical nanoscale aggregates on electrostatic binding of self-assembled multivalent (SAMul) cationic micelles with polyanionic heparin. J. Mater. Chem. B.

[15] Zheng Z., Chen P.Y., Xie M.L., Wu C.F., Luo Y.F., Wang W.T., Jiang J., Liang G.L. (2016). Cell environment-differentiated self-assembly of nanofibers. J. Am. Chem. Soc..

[16] Moore A.N., Hartgerink J.D. (2017). Self-Assembling multidomain peptide nanofibers for delivery of bioactive molecules and tissue regeneration. Acc. Chem. Res..

[17] Fisusi F.A., Notman R., Granger L.A., Malkinson J.P., Schatzlein A.G., Uchegbu I.F. (2017). T-shaped peptide amphiphiles self assemble into nanofiber networks. Pharm. Nanotechnol..

[18] Shi Z.K., Wei Y.H., Zhu C.H., Sun J., Li Z.B. (2018). Crystallization-Driven two-dimensional nanosheet from hierarchical self-assembly of polypeptoid-based diblock copolymers. Macromolecules.

[19] Flood D., Proulx C., Robertson E.J., Battigelli A., Wang S., Schwartzberg A.M., Zuckermann R.N. (2016). Improved chemical and mechanical stability of peptoidnanosheets by photo-crosslinking the hydrophobic core. Chem. Commun..

[20] Sun T.M., Zhang Y.S., Pang B., Hyun D.C., Yang M.X., Xia Y.N. (2014). Engineered nanoparticles for drug delivery in cancer therapy. Angew. Chem..

[21] Xu X.Y., Ho W., Zhang X.Q., Bertrand N., Farokhzad O. (2015). Cancer nanomedicine: From targeted delivery to combination therapy. Trends Mol. Med..

[22] Park K. (2017). New horizons in drug and gene delivery—ICRS 2016. J. Control. Release.

[23] Jain V., Jain S., Mahajan S.C. (2015). Nanomedicines based drug delivery systems for anti-cancer targeting and treatment. Curr. Drug Deliv..

[24] Hancock R.E.W., Sahl H.G. (2006). Antimicrobial and host-defense peptides as new anti-infective therapeutic strategies. Nat. Biotechnol..

[25] Zasloff M. (2002). Antimicrobial peptides of multicellular organisms. Nature.

[26] Qian Y.M., Cui H.Q., Shi R.W., Guo J.N., Wang B., Xu Y., Ding Y.Y., Mao H.L., Yan F. (2018). Antimicrobial anionic polymers: The effect of cations. Eur. Polym. J..

[27] Engler A.C., Wiradharma N., Ong Z.Y., Coady D.J., Hedrick J.L., Yang Y.Y. (2012). Emerging trends in macromolecular antimicrobials to fight multi-drug-resistant infections. Nano Today.

[28] Bechinger B., Gorr S.U. (2017). Antimicrobial peptides: Mechanisms of action and resistance. J. Dent. Res..

[29] Su X.K., Zhou X.Y., Tan Z.Z., Zhou C.C. (2017). Highly efficient antibacterial diblock copolypeptides based on lysine and phenylalanine. Biopolymers.

[30] Zhou X.Y., Su X.K., Tan Z.Z., Zhou C.C. (2018). Synthesis of triblock amphiphilic copolypeptides with excellent antibacterial activity. Eur. Polym. J..

[31] Zhou X.Y., Su X.K., Zhou C.C. (2018). Preparation of diblock amphiphilic polypeptide nanoparticles for medical applications. Eur. Polym. J..

[32] Punia A., Debata P.R., Banerjee P., Yang N.L. (2015). Structure-property relationships of antibacterial amphiphilic polymers derived from 2-aminoethyl acrylate. RSC Adv..

[33] Chakraborty S., Liu R.H., Hayouka Z., Chen X.Y., Ehrhardt J., Lu Q., Burke E., Yang Y.Q., Weisblum B., Wong G.C.L. (2014). Ternary Nylon-3 copolymers as host-defense peptide mimics: Beyond hydrophobic and cationic subunits. J. Am. Chem. Soc..

[34] Liu R.H., Chen X.Y., Chakraborty S., Lemke J.J., Hayouka Z., Chow C., Welch R.A., Weisblum B., Masters K.S., Gellman S.H. (2014). Tuning the biological activity profile of antibacterial polymers via subunit substitution pattern. J. Am. Chem. Soc..

[35] Zhou CC., Wang MZ., Zou KD., Chen J., Zhu YQ., Du JZ. (2013). Antibacterial polypeptide-grafted chitosan-based nanocapsules as an “armed” carrier of anticancer and antiepileptic drugs. ACS Macro Lett..

[36] Zhou X.Y., He J., Zhou C.C. (2019). Strategies from nature: Polycaprolactone-based mimetic antimicrobial peptide block copolymers with low cytotoxicity and excellent antibacterial efficiency. Polym. Chem..

[37] Liu Q.M., Song L.W., Chen S.A., Gao J.Y., Zhao P.Y., Du J.Z. (2017). A superparamagnetic polymersome with extremely high T-2 relaxivity for MRI and cancer-targeted drug delivery. Biomaterials.

[38] Oliveira H., Pérez-Andrés E., Thevenot J., Sandre O., Berra E., Lecommandoux S. (2013). Magnetic field triggered drug release from polymersomes for cancer therapeutics. J. Control. Release.

[39] Chen J., Liu Q.M., Xiao J.G., Du J.Z. (2015). EpCAM-antibody-labeled noncytotoxic polymer vesicles for cancer stem cells-targeted delivery of anticancer drug and siRNA. Biomacromolecules.

[40] Silva M.M., Calado R., Marto J., Bettencourt A., Almeida A.J., Goncalves L.M.D. (2017). Chitosan nanoparticles as a mucoadhesive drug delivery system for ocular administration. Mar. Drugs.

[41] Gibis M., Ruedt C., Weiss J. (2016). In vitro release of grape-seed polyphenols encapsulated from uncoated and chitosan-coated liposomes. Food Res. Int..

[42] Dash S., Murthy P.N., Nath L., Chowdhury P. (2010). Kinetic modeling on drug release from controlled drug delivery systems. Acta Pol. Pharm..

[43] Iwasaki T., Ishibashi J., Tanaka H., Sato M., Asaoka A., Taylor D., Yamakawa M. (2009). Selective cancer cell cytotoxicity of enantiomeric 9-mer peptides derived from beetle defensins depends on negatively charged phosphatidylserine on the cell surface. Peptides.

[44] Kim I., Jin S.M., Han E.H., Ko E., Ahn M., Bang W.Y., Bang J.K., Lee E. (2017). Structure-Dependent antimicrobial theranostic functions of self-assembled short peptide nanoagents. Biomacromolecules.

